# Predicting enhancers in mammalian genomes using supervised hidden Markov models

**DOI:** 10.1186/s12859-019-2708-6

**Published:** 2019-03-27

**Authors:** Tobias Zehnder, Philipp Benner, Martin Vingron

**Affiliations:** Max Planck Institute for Molecular Genetics, Ihnestraße 63-73, Berlin, 14195 Germany

**Keywords:** Enhancer prediction, Epigenetics, Gene regulation, Supervised hidden Markov models

## Abstract

**Background:**

Eukaryotic gene regulation is a complex process comprising the dynamic interaction of enhancers and promoters in order to activate gene expression. In recent years, research in regulatory genomics has contributed to a better understanding of the characteristics of promoter elements and for most sequenced model organism genomes there exist comprehensive and reliable promoter annotations. For enhancers, however, a reliable description of their characteristics and location has so far proven to be elusive. With the development of high-throughput methods such as ChIP-seq, large amounts of data about epigenetic conditions have become available, and many existing methods use the information on chromatin accessibility or histone modifications to train classifiers in order to segment the genome into functional groups such as enhancers and promoters. However, these methods often do not consider prior biological knowledge about enhancers such as their diverse lengths or molecular structure.

**Results:**

We developed *enhancer HMM* (eHMM), a supervised hidden Markov model designed to learn the molecular structure of promoters and enhancers. Both consist of a central stretch of accessible DNA flanked by nucleosomes with distinct histone modification patterns. We evaluated the performance of eHMM within and across cell types and developmental stages and found that eHMM successfully predicts enhancers with high precision and recall comparable to state-of-the-art methods, and consistently outperforms those in terms of accuracy and resolution.

**Conclusions:**

eHMM predicts active enhancers based on data from chromatin accessibility assays and a minimal set of histone modification ChIP-seq experiments. In comparison to other ’black box’ methods its parameters are easy to interpret. eHMM can be used as a stand-alone tool for enhancer prediction without the need for additional training or a tuning of parameters. The high spatial precision of enhancer predictions gives valuable targets for potential knockout experiments or downstream analyses such as motif search.

## Background

The phenotypic variety of cells in eukaryotic organisms across tissues and developmental time is the result of the intricate system of regulation of gene expression. There are many levels on which gene regulation can be achieved, be it on the transcriptional level or on further downstream levels such as post-transcriptional splicing or post-translational modifications. Transcriptional regulation is partly accomplished by the interplay of enhancers and promoters through the activity of transcription factors and has been at the center of research in molecular biology for several decades [1]. Enhancers are thought to clearly outnumber promoters [2, 3] and many genetic diseases are related to mutations in intergenic regions [4, 5], suggesting that the major portion of transcriptional regulation can be attributed to enhancers. However, their characterization and localization has proven to be difficult.

**Fig. 1:**
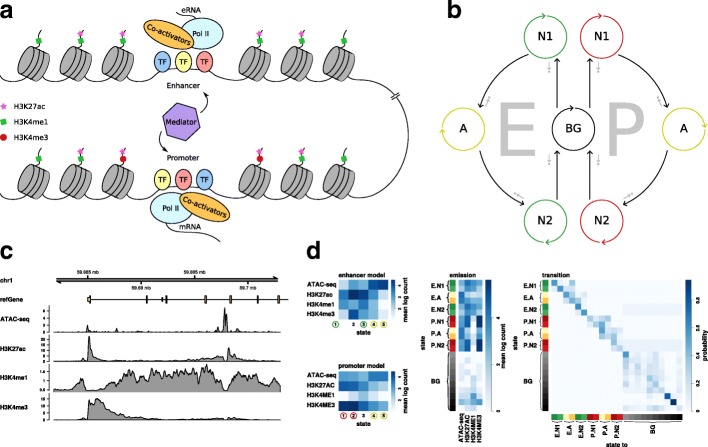
The model. **a** Schematic illustration of the epigenetic environment at enhancers and promoters, derived from [6, 58]. **b** Schematic Markov chain of the underlying constricted Hidden Markov Model. **c** Epigenetic features of an example genomic region. **d** Model parameters. Left: state selection based on emission patterns of the foreground models. Selected states are encircled in green (enhancer nucleosomes), red (promoter nucleosomes), and yellow (accessibility). Right: emission and transition parameters of the full model

In their 2015 review, Heinz et al. [6] describe active enhancers as DNA sequences distal to transcription start sites (TSS) with the potential to elevate basal transcription levels of their target genes. They further describe enhancers as heterogeneous genomic blocks in terms of nucleosome occupation, consisting of a central stretch of accessible, i.e. nucleosome-free DNA and the presence of flanking nucleosomes to both sides. The accessible region provides the contact surface for potential binding events of transcription factors involved in the interaction with the transcription initiation machinery and the recruitment of downstream factors. Chromatin accessibility is experimentally measured by assays such as ATAC-seq [7] or DNase-seq [8]. The flanking nucleosomes delineate the boundaries of the active enhancer and exhibit a distinct pattern of histone modifications such as H3K27ac, H3K4me1 and low levels of H3K4me3 [9, 10]. Studies have shown that enhancers typically co-localize with binding events of the histone acetyltransferase p300 [11–13]. Other features such as unique methylation dynamics [14–16] and bi-directional transcription of so-called enhancer RNA (eRNA) [17] have been described too, and recent efforts in the field of chromatin architecture such as the analysis of spatial chromatin interactions with Hi-C [18] have provided yet another path to capture functional enhancers. A simplified view of the epigenetic environment at enhancers is outlined in Fig. 1a. Figure 1c shows epigenetic signals in an example region around the upstream end of an annotated gene.

Our goal is to integrate available data about enhancer features into a classifier that predicts the genomic locations of enhancers in a genome-wide manner. While some of the experimental methods producing the above-mentioned features are rather laborious, chromatin immunoprecipitation followed by sequencing (ChIP-seq) [19] allows to retrieve the genomic locations of histone modifications in a high throughput manner, making it a widely used technique in many laboratories. Thus, many computational enhancer prediction methods have been developed that use histone modification ChIP-seq data as input. These methods fall into two classes: unsupervised methods that do not include prior biological knowledge and require the user to interpret the predictions, and supervised methods that rely on a set of positive samples to train on, thereby yielding predictions that reflect the properties of the training set. Many mathematical models have been employed in both unsupervised and supervised manner (see [20, 21] for review), one of the most prominent ones is the hidden Markov model (HMM) [22].

HMMs can be used to infer an unknown state associated with each position in a given sequence of observations. They assume that observations are generated by an underlying hidden state emitting symbols according to a particular probability distribution. HMMs are therefore ideal for the task of recognizing chromatin states based on the observed sequence of histone modification patterns, and have repeatedly been used for that purpose in an unsupervised, as well as a supervised fashion. Chromatin annotation methods such as ChromHMM, EpiCSeg or Genostan [23–25] implement an unsupervised HMM, i.e. the main hyperparameter is the desired number of states. These methods require the user to interpret and annotate the learned states based on previous knowledge about functional elements in the genome, e.g. that promoters are enriched in H3K4me3 signal. Won et al. [26] turn this approach around and use supervised HMMs with a leftright structure to predict different genomic modules such as enhancers, promoters and background, and incorporate the modules into one model. They integrate existing knowledge into the model by learning the parameters on preselected training sets. However, their model allows the modules to be passed through in many different ways, e.g. skipping the state representing the nucleosome-free region where transcription factors can bind, leaving the method very sensitive for detecting false positives. Unfortunately, we were not able to test their method as the software is not available. Other methods rely on different mathematical models in order to predict enhancers [27–29], and many of them do not consider prior biological knowledge about enhancers such as their diverse lengths.

To address this, we designed *enhancer hidden Markov model* (eHMM), a supervised hidden Markov model consisting of three modules, each being learned on a designated training set for enhancers, promoters, and background, respectively. As promoters and enhancers exhibit a substantial overlap in histone modification patterns, this distinction helps the enhancer model not to primarily detect annotated promoters. We acknowledge recent reports attributing enhancer function to some promoters [30], however, this dual role is not within the scope of this article. eHMM implements enhancer and promoter models reflecting the physical structure comprising a central accessible stretch of DNA flanked by two nucleosomes. The enhancer and promoter modules, subsequently referred to as the foreground modules, can only be reached through transitions from the background module to a state representing the first nucleosome (Fig. 1b). Aside from self-transitions, that state can only be left for a chromatin accessibility state and from there further to the second nucleosome and back to the background module. This imposition of specific state transitions confers the desired topology on the foreground modules.

In the following sections we describe the method, compare the performance of eHMM to both unsupervised and supervised methods within and across cell types and show that eHMM outperforms previous methods in prediction accuracy and resolution. Based on measuring the area under the precision-recall curve, eHMM performs at levels comparable to state-of-the-art methods. Moreover, eHMM is easy to interpret, yields predictions with a high resolution and provides a pre-trained model that can robustly be applied across samples.

## Results

We developed eHMM in order to identify enhancers throughout the genome. The model is designed to capture an enhancer’s topology, consisting of a central accessible stretch of DNA flanked by two nucleosomes (see Methods). Chromatin accessibility is measured with the DNA accessibility assay ATAC-seq. Nucleosomes are detected from the occurrence of ChIP-seq signals for the three histone modifications H3K27ac, H3K4me1 and H3K4me3. H3K27ac is generally associated with active chromatin, whereas ratios of H3K4me1 over H3K4me3 are typically high at enhancers and low at promoters. This small set of four features provides a maximal amount of information while being minimally redundant at the same time. Moreover, it consists of only the most prevalent histone marks for which antibodies are available for many species. In this section we discuss the performance of eHMM within and across cell types and developmental stages, compare it to state-of-the-art methods and study the features of called enhancers and promoters.

### Cross validation of enhancer predictions

The ENCODE consortium provides an extensive catalog of functional genomic data including numerous ChIP-seq experiments across many organisms, tissues, cell types, developmental stages and treatments [3]. We use ChIP-seq data for the histone modifications H3K27ac, H3K4me1 and H3K4me3, as well as ATAC-seq data to train the method on. The FANTOM consortium provides CAGE data for many of these tissue-stages [31], enabling us to establish respective training sets on features orthogonal to the histone modification ChIP-seq and ATAC-seq used for learning. Together, these data sets allow us to test our method and compare it to state-of-the-art software.

**Fig. 2:**
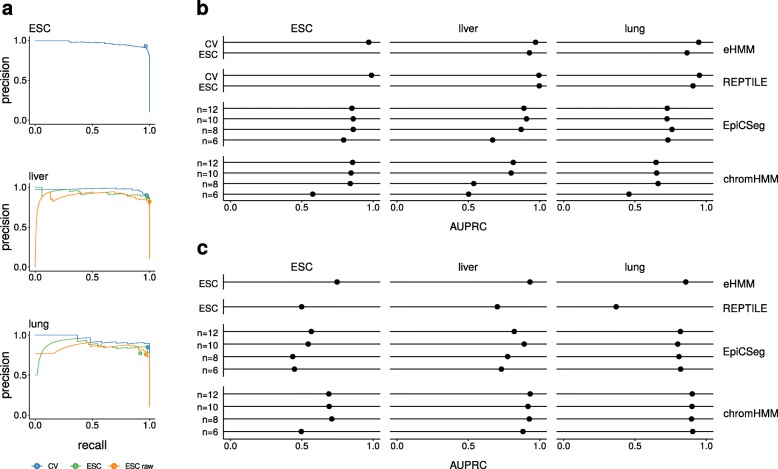
Validation. **a** Precision recall curves of eHMM in within and across sample validation schemes on the FANTOM5 data in mouse ESC, liver and lung. Circles indicate prediction performance of the viterbi algorithm, while the lines represent precision and recall based on posterior probabilities obtained from the forward-backward algorithm. **b-c** Comparison of areas under precision recall curve using different enhancer prediction methods validated on regions from FANTOM5 (**b**) and Enhanceratlas (**c**). Legend acronyms: CV - within-sample 5-fold cross-validation. ESC - across-sample validation using a model trained on ESC data including quantile normalization. ESC raw - across-sample validation using a model trained on ESC data without normalization. n - number of states

We performed a 5-fold cross-validation scheme on three different mouse samples (ESC E14, liver E12.5, lung E16.5). We created unbalanced training and test sets with the aim to reflect genomic proportions as described in the “Methods” section, such that each test set contains 1/5 of the original enhancer training set. eHMM is able to recall a very high fraction of the FANTOM5 enhancers without capturing a lot of false positives, i.e. being very precise at the same time, depicted by a sample-specific area under the precision-recall curve (AUPRC) of 0.947 - 0.971 (Fig. 2a). Notably, even low threshold values yield high precision while still capturing most enhancers from the test set.

Often, enhancer predictions are desired in specific samples for which it is unfeasible to define a training set. Thus, it is necessary to be able to train the method on one sample and apply it to another. We tested eHMM’s performance in cross-sample validation settings where we used the model trained on ESC to predict FANTOM5 enhancers in liver E12.5 and lung E16.5. We used quantile normalization (see Methods) to account for potentially different read count scales between samples. As expected, method performance decreases slightly in across-sample validation compared to using a model trained on data from the same sample. Areas under the precision-recall curve of 0.928 and 0.865 for liver E12.5 and lung E16.5, respectively, still show very satisfying results. This demonstrates the method’s great applicability with pre-trained models. Moreover, we show the suitability of the quantile normalization approach by comparing cross-sample validations with and without normalization. Normalization helps to improve prediction quality with an increase in area under the precision-recall of 0.041 and 0.025 in liver E12.5 and lung E16.5, respectively.

### Comparison to existing methods

Numerous software packages exist for predicting regulatory elements, relying on various experimental data [20, 21]. In this subsection we compare the prediction performance of our method to ChromHMM [23], EpiCSeg [24] and REPTILE [32]. We chose these methods for a variety of reasons. First, ChromHMM is a well-established and widely used method that learns a hidden Markov model based on binarized input data in an unsupervised fashion. EpiCSeg presents another unsupervised HMM that also provided the foundation of the implementation of eHMM. In contrast to ChromHMM, it models the read count data using a negative multinomial distribution instead of binarized data. Together, these two methods allow us to compare our supervised HMM to two unsupervised HMMs and thus to investigate the benefit of supervision. Finally, REPTILE is a supervised method using a random forest classifier, which we train with the same training data as eHMM in order to study the differences between two supervised methods. As shown in their article [32], He et al.’s REPTILE outperforms many previous methods and therefore certainly serves as a challenging competitor to eHMM.

ChromHMM and EpiCSeg were applied to whole genome data with different numbers of states (6, 8, 10 and 12). We computed the maximum posterior probability of every state in the test regions and report only the best performing state. REPTILE and eHMM were tested within cell types using 5-fold cross-validations on FANTOM5 data and across cell types by validating the performance of a model trained on mouse ESC on enhancer regions from FANTOM5 and EnhancerAtlas [33].

**Within cell type validation** Figure 2b shows a comparison of the AUPRC for predictions with eHMM, REPTILE, ChromHMM and EpiCSeg in three different cell types. The unsupervised methods ChromHMM and EpiCSeg were trained with different numbers of states *n* and in most cases tend to perform best with *n*=10 or *n*=12. The supervised methods eHMM and REPTILE performed very similarly, with both of them clearly outperforming ChromHMM and EpiCSeg and thus demonstrating the benefit of supervised learning.

**Cross cell type validation** In order to test the supervised methods’ performance across cell types, we applied ESC-trained models to samples from different cell types. We first tested their ability to predict the previously defined FANTOM5 enhancers for liver E12.5 and lung E16.5. Consistently, eHMM and REPTILE achieve higher prediction accuracy than ChromHMM and EpiCSeg (Fig. 2b).

In addition, we compared the methods’ performance on regions from the EnhancerAtlas for cell types ESC E14, liver E14.5 and lung E14.5 (Fig. 2c). It is notable that all methods perform better in lung and liver compared to ESC. In all cell types, eHMM and ChromHMM perform best. REPTILE struggles with this setting, possibly due to overfitting of the learned models on the FANTOM5 data. These results underline the robustness of eHMM under different types of validation setups.

### Whole genome enhancer predictions in mouse ESC

We used eHMM for a genome wide search for enhancers in mouse embryonic stem cells. The model returns the most likely global path (see “Methods” section), resulting in the prediction of 5357 enhancers and 8040 promoters without the need to select a prediciton threshold. Depending on the prediction threshold *c*, REPTILE predicts between 2604 (*c*=0.9) and 12,830 (*c*=0.1) enhancers. Varying the number of states *n*, ChromHMM finds between 19,643 (*n*=12) and 88,716 (*n*=6) enhancers, EpiCSeg between 37,911 (*n*=12) and 103,293 (*n*=6).

**Fig. 3:**
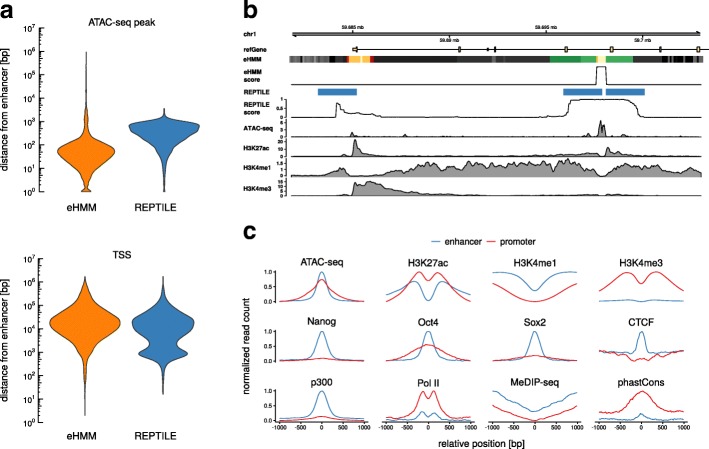
Whole genome predictions in mouse ESC. **a** Mean feature distributions of predicted enhancers and promoters in mouse ESC. **b** Example genomic region with predictions from eHMM and REPTILE (threshold = 0.5). The color code in the eHMM segmentation track is equal to Fig. 1c. **c** Distance distributions of predicted enhancers to closest ATAC-seq peak (MACS2) and TSS (UCSC knownGene database) in mouse ESC for eHMM and REPTILE (threshold = 0.9)

In the remaining subsection we discuss the properties of eHMM’s predicted enhancers and promoters in mouse ESC as depicted in Fig. 3a.

**Histone modifications** The identified regulatory regions exhibit the anticipated presence or absence of particular histone modifications, e.g. predicted enhancers show on average higher levels of H3K4me1 than promoters, while in turn promoters exhibit higher levels of H3K4me3. Notably, all histone modifications show a distinct bimodality while transcription factor binding events are unimodally distributed with centered peaks, providing evidence for our initial biological assumption.

**Binding of transcription factors and chromatin remodelers** Further, predicted enhancers show enriched binding of ESC specific transcription factors Nanog, Oct4 and Sox2. It is worth noting that these lineage-specific transcription factors are enriched more strongly in predicted enhancers compared to promoters, in line with the hypothesis that enhancers are more lineage-specific than promoters, and that promoters can be regulated by different sets of lineage-specific enhancers depending on the cell type [34]. In addition, predicted enhancers show elevated levels of the histone acetyltransferase p300, an enzyme involved in transcriptional regulation via chromatin remodeling and associated with active enhancers [13]. Binding events of CCCTC-binding factor (CTCF), a protein involved in the regulation of the three dimensional chromatin structure [35] and often co-occurring with the borders of topologically associated domains, are enriched in enhancers, implying the enhancers’ role in the mediation of enhancer-promoter contacts and DNA looping [36, 37].

**DNA methylation and sequence conservation** Both enhancers and promoters show a dip in DNA methylation measured by MeDIP-seq. This effect appears to be stronger in predicted promoters, confirming recent studies that suggest that DNA methylation levels negatively correlate with H3K4me3 [16] and are low at promoters in general [14]. Promoters exhibit increased sequence conservation across species as measured by phastCons. Enhancers indicate this feature as well, but to a much lower extent, confirming previous reports [38, 39].

**RNA Polymerase II** Finally, promoters exhibit high levels of RNA Polymerase II, indicating transcription initiation events. Enhancer elements show a similar pattern but at lower levels, confirming that the input data from FANTOM5 reflects the information about the bidirectional transcription initiation which had originally motivated our choice of the training set.

### Spatial accuracy of predictions

In addition to the reassuring properties of the predicted enhancer regions, eHMM also provides predictions that are spatially highly accurate, because the model distinguishes between nucleosomal and accessible states. We assessed the spatial accuracy of predicted enhancers using the distances of their centers to the closest ATAC-seq peak. We used a prediction threshold of 0.9 for REPTILE as this produced lowest distances. eHMM predictions are on average around eight times closer to the center of an accessible region compared to REPTILE (median of 42 bp and 343 bp, respectively, Fig. 3c). Other features such as DNA methylation might improve REPTILE’s spatial prediction accuracy, however, at the expense of requiring additional data.

### False enhancer predictions near promoters

Promoters and enhancers are mainly distinguished by the degree of methylation of lysine 4 at histone 3. Promoters generally show strong H3K4me3 signals in the immediate proximity to their center. Moving away from a promoter’s center, this signal usually decreases fast and H3K4me1 levels rise, resembling the nucleosomes of a typical enhancer. However, these nucleosomes are in the periphery of promoters and do not border accessible chromatin. Figure 3b illustrates this problem, showing an example gene where eHMM correctly predicts a promoter at the upstream end of a transcribed gene, while REPTILE misclassifies the adjacent region as an enhancer. We quantified this effect by calculating the fraction of genome-wide predicted enhancers that overlap an annotated TSS. Depending on the prediction threshold *c*, the fraction of enhancers predicted by REPTILE that overlap an annotated TSS ranges from 17.8% (*c*=0.9) to 35.0% (*c*=0.2), whereas this measure is 3.2% for enhancers predicted by eHMM. Distances of predicted enhancers to the closest annotated TSS are unimodally distributed in the case of eHMM with an interquartile range spanning from 11 kb to 85 kb (Fig. 3c). Enhancers predicted by REPTILE exhibit an additional mode that centers at approximately 1 kb.

### Run times

We estimated empirical run times for model training and prediction on mouse ESC data and compared them to those of REPTILE, EpiCSeg and ChromHMM. All methods ran on 21 cores in parallel as far as the respective implementation allowed it. Run times per core are shown in Table 1. REPTILE uses the least total CPU time, but the longest real time, indicating a lack of efficiency in leveraging multithreading.

**Table 1:** Run times

	Real time [s]			CPU time [s]		
Method	Training	Prediction	Total	Training	Prediction	Total
eHMM	2.961	43.636	46.597	15.337	155.820	171.157
REPTILE	1.461	89.456	90.917	5.162	140.388	145.550
EpiCSeg			36.327			352.294
ChromHMM			50.401			282.909

## Discussion

We developed an enhancer hidden Markov model called eHMM with the goal of detecting enhancers with variable lengths throughout mammalian genomes. eHMM features three sub-models for enhancer, promoter and background, each being trained in a supervised fashion on predefined training sets. The enhancer and promoter models consist of a particular architecture that captures the biological topology of these regulatory elements, i.e. a central accessible stretch of DNA flanked by nucleosomes to each side.

Our method performs very well in cross-validation tests (AUPRC > 0.94, Fig. 2a), showing that the proposed physical model is present in the data and captured by eHMM. Moreover, eHMM incorporates a quantile normalization step that makes it well applicable across samples, e.g. a model trained on one cell type or developmental stage can be used for predictions on another. Based solely on the area under the precision-recall curve as a performance measure, eHMM achieves similar results as the top-performing state-of-the-art software REPTILE when testing on the FANTOM5 data set, and outperforms it when validating on regions from the EnhancerAtlas. These results suggest overfitting of the models learned by REPTILE and underline the robustness of eHMM’s predictions over different validation setups. Notably, there are apparent performance differences between cell types, in particular the prediction performance on ESC is generally lower compared to lung and liver. This is likely due to the fact that EnhancerAtlas regions were predicted on the basis of agreement of different source tracks such as TFBSs, eRNA, histone modifications, chromatin accessibility and more. Here, we use only chromatin accessibility and histone modifications, and we would thus expect the tested methods to perform best in cell types where these features were most informative for the EnhancerAtlas predictions. The results suggest that ESC regions in the EnhancerAtlas were not primarily predicted on the basis of the features used in this study.

The outcome of unsupervised methods such as ChromHMM and EpiCSeg is uncertain as they perform well in some conditions and poorly in others, and it is not apparent how to judge the quality of a segmentation without a test set. In addition, state interpretation is not trivial and highly affects the prediction quality.

Genome-wide detected enhancers and promoters in mouse ESC exhibit expected properties, confirming prediction quality. For example, lineage-specific transcription factors are enriched at enhancers, and promoters exhibit low DNA methylation levels and an abundance of RNA Polymerase II. In contrast to previous work focusing on sequence conservation in cis-regulatory regions [40, 41], our results show that the sequence of predicted enhancers is less conserved in comparison to predicted promoters. This seeming contradiction between observing strong binding of lineage-specific transcription factors and low levels of sequence conservation could suggest functional conservation while the enhancers’ genomic locations are highly dynamic in evolutionary terms as suggested by Schmidt et al. [38], manifesting itself in a lower sequence conservation across species. The lower number of predicted enhancers with the supervised methods eHMM and REPTILE reflects their higher specificity compared to the unsupervised methods ChromHMM and EpiCSeg. While REPTILE enforces this specificity rather arbitrarily by calling only the most certain enhancer among multiple neighboring predictions, eHMM achieves this by the potential presence of enhancer- and promoterlike states in the background model that compete with the topology-respecting foreground model. eHMM thus ultimately reduces the false-positive rate by emphasizing the importance of the enhancers’ molecular structure, which in turn results in higher spatial accuracy (see example in Fig. 3b). Further, eHMM returns the most likely path according to the Viterbi decoding algorithm and therefore does not require the definition of an arbitrary prediction threshold.

REPTILE often predicts enhancers right next to promoters where the promoter-specific histone modification H3K4me3 decreases while H3K4me1 remains. The implemented promoter model as well as the aforementioned model topology enables eHMM to distinguish between the two regulatory elements and to refrain from calling enhancers in promoter-associated regions merely on the basis of a decreasing promoter signal.

In addition, eHMM provides a high resolution of predicted regions, allowing to accurately target regulatory subunits such as nucleosomal or accessible regions for potential downstream analyses. Moreover, eHMM allows inspection of model parameters that provide information about both transition dynamics between states and each state’s signal emission distribution, standing in contrast to “black box” methods such as random forests. These properties facilitate interpretability of the learned parameters and the predicted regions.

Finally, we show how to use hidden Markov models in a supervised fashion with genomic data, and how different models learned on various training sets can be combined in order to obtain one global model containing supervised modules with well-defined topologies.

Taken together, the minimal feature requirements, good performance within and across samples, the predictions’ high spatial accuracy as well as interpretability and resolution makes eHMM a very powerful and feasible tool for enhancer prediction.

## Conclusion

In summary, we have presented enhancer hidden Markov model (eHMM), which predicts enhancers based on data from histone modification ChIP-seq and chromatin accessibility assays. eHMM is easy to use since it does not require user decisions such as state examination or the choice of a prediction threshold, and it comes with a pre-trained model as well as the option to let it learn a model on self-designed training sets.

## Materials & methods

### Data types

We used data from chromatin immunoprecipitation followed by sequencing (ChIP-seq) experiments for histone modifications (HM) and transcription factors (TF). ChIP-seq uses protein-specific antibodies to isolate DNA that physically interacts with the protein of interest. Chromatin accessibility was studied using data from an Assay for Transposase Accessible Chromatin using sequencing (ATAC-seq). ATAC-seq uses hyperactive prokaryotic transposase T5, an enzyme that targets accessible DNA in a sequence-unspecific manner.

We investigated five specific cell types, i.e. mouse embryonic stem cells E14 (ESC), mouse embryo liver E12.5 and E14.5 and mouse embryo lung E14.5 and E16.5. ATAC-seq and HM ChIP-seq data from liver and lung samples were obtained from ENCODE [3]. We downloaded ESC HM and TF ChIP-seq and Methylated DNA immunoprecipitation followed by sequencing (MeDIP-seq) data from Gene Expression Omnibus (GEO) [42], and converted genome coordinates from mm9 to mm10 with crossmap [43]. We obtained sequence conservation data using phastCons conservation scores from UCSC [44]. An overview of all used data and their accession numbers is given in Table 2.

**Table 2:** Data sources. Accession numbers containing GSE were obtained from GEO [59–62], those starting with ENC from ENCODE

Cell type	Experiment	Target	Accession	Format
ESC E14	ATAC-seq	-	GSE120376	fastq
	ChIP-seq	H3K27ac	GSE120376	fastq
		H3K4me1	GSE120376	fastq
		H3K4me3	GSE120376	fastq
		Nanog	GSE11431	fastq
		Oct4	GSE11431	fastq
		Sox2	GSE11431	fastq
		CTCF	GSE29184	fastq
		p300	GSE29184	fastq
		Pol II	GSE29184	fastq
	MeDIP-seq	-	GSE3859	fastq
liver E12.5	ATAC-seq	-	ENCSR302LIV	bam
	ChIP-seq	H3K27ac	ENCSR136GMT	bam
		H3K4me1	ENCSR770OXU	bam
		H3K4me3	ENCSR471SJG	bam
liver E14.5	ATAC-seq	-	ENCSR032HKE	fastq
	ChIP-seq	H3K27ac	ENCSR075SNV	bam
		H3K4me1	ENCSR234ISO	bam
		H3K4me3	ENCSR433ESG	bam
lung E14.5	ATAC-seq	-	ENCSR335VJW	fastq
	ChIP-seq	H3K27ac	ENCSR452WYC	bam
		H3K4me1	ENCSR825OWH	bam
		H3K4me3	ENCSR839WFP	bam
lung E16.5	ATAC-seq	-	ENCSR627OCR	fastq
	ChIP-seq	H3K27ac	ENCSR140UEX	bam
		H3K4me1	ENCSR387YSD	bam
		H3K4me3	ENCSR295PFM	bam

### Data processing

We downloaded the raw data fastq files using the SRA toolkit [45] and processed fastq to bam files using the Burrows-Wheeler Alignment tool (BWA) [46] for mapping and SAMtools [47] for filtering, sorting and removing duplicates. eHMM implements the algorithm *bamsignals* [48] to calculate read counts for bins with a width of 100 bp. In order to estimate the fragment centers and with an expected fragment length of 150 bp, bamsignals adds a default shift of 75 bp to ChIP-seq reads. In contrast, chromatin accessibility assays are treated with a shift of zero as the interest of these experiments lies on the actual cutting sites. We added a pseudo-count of 1 to prevent taking logarithms of entries with value zero (see “eHMM algorithm” subsection).

Data from different ChIP-Seq experiments may vary in their total number of reads and their read count distributions may be scaled differently. Therefore, in order to apply a model learnt on a specific cell type to another cell type, input data has to be brought to the same scale. We used quantile normalization to adjust the statistical properties of a query distribution (the data the model is applied to) to a reference distribution (the data the model was learned on) [49]. This method minimizes the distance between the query and reference cumulative distributions by an order-preserving rescaling of the query count values.

### Training regions

To date, there is no gold standard set of true enhancers. However, there is a plethora of experimental approaches for identifying enhancers [31, 50]. Since the model learns patterns of ATAC-seq and HM ChIP-seq signals, we defined the training set based on criteria independent of HM ChIP-seq. FANTOM5 is a project of the FANTOM consortium that uses Cap Analysis of Gene Expression (CAGE) sequencing on RNA samples in order to detect short abortive bi-directional transcription events throughout the genome [31]. We applied the following protocol to the publicly available CAGE data sets for mouse embryonic stem cells E14, liver E12 and lung E17 in order to define our enhancer training regions:

We set a minimal threshold of 11 (ESC) and 5 (liver, lung) CAGE-tags per region resulting in 5573, 537 and 642 regions, respectively. We performed k-means clustering on the regions’ ATAC-seq, H3K27ac and H3K4me1/3 ChIP-seq signals with *k*=5 and selected the cluster with the strongest active enhancer signature consisting of 920 regions in ESC. The discarded clusters exhibited typical patterns of promoters, poised enhancers, or were depleted of any signal. The model topology requires the training regions to be accurately defined, i.e. to start and end at nucleosome positions. To that end, we used MACS2 [51] with default settings to determine H3K27ac - ATAC-seq - H3K27ac peak triplets with a width of less than 2 kb overlapping with the active enhancer regions, followed by the removal of neighboring regions (pairwise distance of less than 2 kb). This procedure resulted in a set of 647 active enhancer regions in ESC, from which 300 regions were sampled randomly. We applied the same procedure to annotated promoters from the UCSC knownGene database [52]. From the resulting 3029 regions with a H3K27ac signal above the minimum of the previously defined active enhancer regions, 300 were randomly sampled to give rise to the training set for the ESC promoter model. Training sets for liver and lung were obtained analogously.

**Fig. 4:**
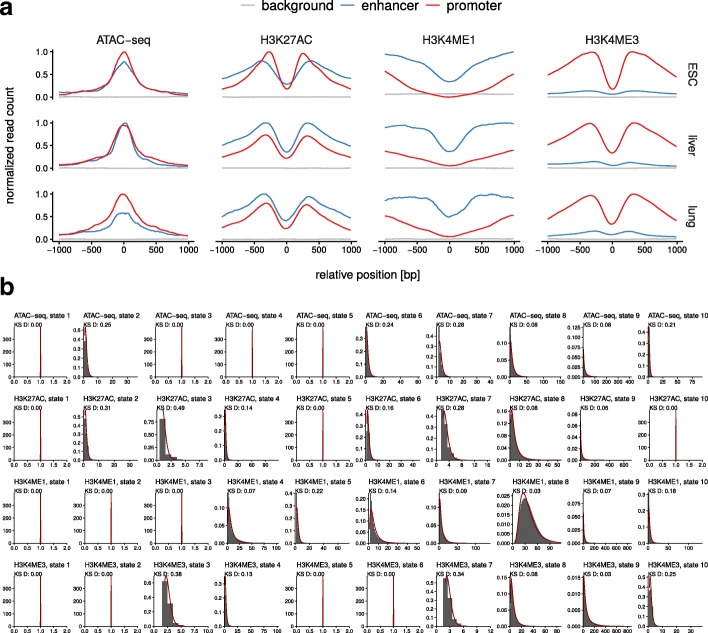
Read counts. **a** Distribution of normalized read counts for training regions of mouse ESC E14, mouse embryonic liver E12.5 and mouse embryonic lung E16.5. **b** Histograms of read count data (grey) and fitted log-normal distributions (red) of an unsupervised 10-state HMM learned on whole genome ESC data

In order to define a background training set representing everything except enhancers and active promoters, we defined the proportions of functional elements in mammalian genomes by roughly approximating the numbers reported for the human genome by Kellis et al. [53]. This resulted in 10% enhancers, 5% active promoters, 5% inactive promoters, 10% genic and 70% intergenic regions. The training set for the background model was obtained by randomly sampling 2 kb genomic regions according to these proportions with respect to UCSC knownGene annotations, leaving out regions annotated as enhancers or active promoters. Figure 4 shows the average signal distributions for the enhancer, promoter and background training regions in all three cell types.

### Test regions

We used the previously described training regions in ESC, liver E12.5 and lung E16.5 for cross-validation as well as cross cell type validation. In addition, we defined test sets in ESC, liver E14.5 and lung E14.5 using regions from the EnhancerAtlas [33]. We processed the data sets by combining regions within 500 bp, excluding regions that are located within 2 kb of annotated promoters from the UCSC knownGene database and centering on the highest overlapping ATAC-seq peak in order to emphasize our intention to focus on functional enhancers. Notably, this led to data set reductions of 68%, 83% and 66% for ESC, liver and lung, respectively. We complemented the test sets with randomly sampled regions according to the proportions of functional elements in mammalian genomes with respect to UCSC knownGene annotations.

### eHMM algorithm

**Probabilistic model** Our method eHMM implements a probabilistic framework based on a multivariate HMM [22, 54] with specific constraints. HMMs are used to model a series of observations emitted by a sequence of *n* distinct *hidden* states. An HMM is characterized by the *n*×*n* transition matrix containing the probabilities of moving between states and a set of emission distributions defining the probability by which a particular state emits an observation. Standard HMMs are unsupervised and typically learn the transition and emission parameters for a given number of states using the Baum-Welch algorithm [22].

Our approach differs from a conventional HMM in that it is built from three parts: an enhancer model, a promoter model (in combination referred to as the foreground model) and a background model. The key characteristic of both foreground models is directionality, as depicted in the corresponding Markov chain in Fig. 1b: Both enhancer (E) and promoter (P) models can only be reached through transitions from the background (BG) to states representing the first nucleosome (N1), from which accessible-chromatin states (A) and later a second nucleosome state (N2) have to be visited before returning to BG. In addition, self-transitions allow the model to capture regulatory elements of variable lengths.

All three sub-models are learned in a supervised manner on predefined training sets. For the enhancer and promoter models, this is achieved by a two-step learning process. First, a conventional 5-state HMM is learned on the training set, followed by a state selection step where states are assigned to represent either accessibility (A-states) or nucleosome (N-states) based on their emission parameters (see example in Fig. 1c). The automated state selection assigns the two states with the highest ATAC-seq/H3K27ac (or DNase-seq/H3K27ac) ratio to A. From the remaining three states, the two with the highest (enhancer model) or lowest (promoter model) H3K4me1/H3K4me3 ratio are selected as N-states. The ratios are calculated on the mean of the fitted log-normal distributions. Then, N-states are duplicated to N1 and N2 and arranged in a directed order together with the A-states. Transitions conflicting with the directionality, e.g. from N2 back to A, are forbidden by setting the corresponding transition probabilities to zero. See Fig. 1b for illustration.

We use Viterbi training [55, 56] instead of the Baum-Welch algorithm, which allows to force the regions to end in a N2-state. Viterbi training is a simplification of the Baum-Welch algorithm and its result is an approximation of the maximum likelihood estimate. Instead of accounting for all possible paths, only the most probable path is considered during parameter re-estimation. In addition, during Viterbi training we only allow the transition parameters to change while emission parameters are fixed, thereby preventing states previously assigned to a particular class to adapt [57]. With these constraints we hope to achieve an accurate representation of enhancer and promoter characteristics reflected by both emission and transition parameters.

The background model is a conventional 10-state HMM learned on a predefined unbalanced training set that represents the aforementioned proportions of functional elements in mammalian genomes.

Next, the three sub-models are combined into one model consisting of all states (see example in Fig. 1c). Transitions between states of different sub-models are either set to zero because they are not allowed, or estimated in the case of BG-N1 or N2-BG transitions. For the first, we refer to the estimated number of enhancers (399,124) and promoters (70,292) in the human genome as stated by the ENCODE consortium [3], as well as to the total human genome size of roughly 3 billion bp according to genome assembly GRCh38, and a bin size of 100 bp. These numbers lead to estimated BG-N1 transition rates of 1.33% and 0.23% for enhancers and promoters, respectively, and we expect them to be good estimates for other mammalian genomes, too. We set N2-BG transitions to the learned values of N1-A transitions as the sizes of N1 and N2 are expected to be equal.

The algorithm is incorporated into the EpiCSeg framework [24] and offers the user the choice between learning a model from given training sets or using the provided pre-trained model, whose learned parameters are discussed in “Results” section.

**Emission distributions** Mammana et. al [24] show that multivariate read count data can be accurately modeled using the negative multinomial distribution. However, the fitting procedure for negative multinomials requires a complex numerical approximation. Instead, we fitted the read count data with independent log-normal distributions, which appear to be both a better fit for the data as well as the analytical fitting procedure being much easier. Fit quality is demonstrated in Fig. 4b, showing the read count data and the fitted log-normal distributions in an unsupervised 10-state model learned on whole genome ESC data. Kolmogorov-Smirnov (KS) distances between the data and the fits were computed for all features and states, ranging from 0.00 to 0.49 with a median of 0.08. Some components model a single coverage value and we assume here that such states have a KS distance of 0. In contrast, marginal negative binomial fits show KS distances ranging from 0.02 to 0.29 with a median of 0.09 (data not shown).

**Decoding and scoring** There are several decoding algorithms that yield a state sequence from a learned HMM. Posterior decoding determines the path with the most probable state at any time point. However, it may not preserve the model’s grammar, which is essential in order to prevent forbidden transitions e.g. from a state representing an accessible region to a background state. Hence, we use the Viterbi decoding algorithm, which returns the globally most likely path, resulting in a particular number of predicted enhancers without the requirement for finding an optimal prediction threshold. However, while these predictions all belong to the globally most likely path, they might differ in local certainty. The posterior decoding algorithm provides a posterior probability for the respective state at each position, considering all possible paths. Summing over the posteriors of the states representing accessibility at every position provides a measure of prediction certainty with expected maxima at the center of predicted enhancers.
